# Cone beam CT in the imaging of musculoskeletal trauma: a scoping review

**DOI:** 10.1007/s00256-025-04947-w

**Published:** 2025-05-14

**Authors:** Jessica R. Smith, Balvinder K. Bharath, Martine A. Mallinson, Kim Mason, Beverly Snaith

**Affiliations:** 1 Mid Yorkshire Teaching NHS Trust, Aberford Road, Wakefield, West Yorkshire WF1 4DG UK; 2 Keele University, Staffordshire, ST5 5BG UK; 3 University of Leeds, Woodhouse, Leeds, LS2 9 JT UK; 4 University of Bradford, Bradford, West Yorkshire BD7 1DB UK

**Keywords:** Cone beam computed tomography, Imaging, Trauma, Musculoskeletal, Occult fracture

## Abstract

**Introduction:**

Cone beam computed tomography (CBCT) is an emerging technology in musculoskeletal (MSK) imaging. The objective of this scoping review was to provide an overview of the research surrounding CBCT utility in bony injury assessment as an alternative to other imaging modalities and investigate any gaps in the current evidence base.

**Methods:**

MEDLINE, CINAHL, and PubMed were searched up to January 2025 for articles including CBCT studies on human participants following trauma. An online literature review tool was used to manage and streamline the review process.

**Results:**

The search yielded 23 studies. The image quality and diagnostic accuracy of CBCT were high overall, and a number of studies confirmed the radiation dose to be lower than multislice CT. Studies examined CBCT for extremity trauma, with half the studies focused solely on the wrist. The utility appears greatest in the identification of radiographically occult fractures. Limited cost-effectiveness analysis has been undertaken.

**Conclusions:**

Overall, the literature suggests CBCT can be an effective tool in the diagnosis of bony injuries with greater sensitivity than radiography at a lower radiation dose than multi-slice computed tomography. However, evaluation of wider patient and economic impacts of adopting CBCT in MSK trauma pathways is recommended.

## Introduction

Cross-sectional imaging plays a key role in the diagnosis and treatment planning of patients with musculoskeletal (MSK) trauma [1], whereas multi-slice computed tomography (MSCT) is established in the field and cone beam computed tomography (CBCT) is an emerging application [2, 3]. CBCT has been utilized in orthodontics and maxillofacial imaging for a number of years [4–6] but has recently evolved to include the imaging of a range of other anatomical regions and pathologies [7]. Essentially, CBCT is a three-dimensional (3D) imaging modality that uses a rotating gantry with a fixed pulsating conical X-ray beam and, usually, a flat panel detector [8]. This mode of acquisition allows multiplanar and cross-sectional images to be produced, which are considered comparable in imaging characteristics to MSCT [9–11]. Although the resultant images are similar, the CBCT technology means the systems are smaller and, depending on the scanner type, can be relocatable or adaptable to different patient groups [12]. Some CBCTs can also be used with patients seated, supine, or standing, enabling weight-bearing or non-weight–bearing images to be acquired.

Because of the simpler technology, the scanners are lower in initial purchase than MRI and MSCT. CBCT also has a smaller footprint, which means it can be installed in more confined spaces and uses less energy than other 3D technologies without the need for the usual three-phase electrical supply, resulting in lower running costs [3]. With potential MSK applications spanning diagnostic and interventional procedures across acute and chronic disease, CBCT is still yet to be widely implemented in clinical practice globally, and as such, the true potential impact of the technology is unclear [13]. One of the key opportunities widely reported is in the assessment of bony injuries [7, 12–14], and this scoping review examines the literature pertaining to CBCT use within the trauma context and aims to consider the current evidence for its utilisation.

## Materials and methods

This scoping review complied with and was guided by the Joanna Briggs Institute (JBI) manual for evidence synthesis [15] and was conducted in accordance with the Preferred Reporting Items for Systematic Reviews and Meta-Analysis (PRISMA) checklist [16]. A scoping review was selected as the best approach as it enables evidence identification and mapping of the literature within a specific context without restriction [17–20].

### Search strategy

In line with the scoping review methodology [15] and similar research [21], a wide search strategy was employed to identify literature and technology-based terms, including “cone beam computed tomography,” “weight-bearing tomography,” and “X-ray computed tomography” were used.

The databases MEDLINE, CINAHL, and PubMed were searched in February 2023 with the search repeated in July 2024 and January 2025 to identify any additional new publications. Limitations were applied to include only studies published over the last 15 years in the English language. Data were subsequently imported to Covidence (Veritas Health Innovation, Melbourne, Australia), a web-based review platform to complete screening, review, and data extraction [22].

### Study selection

The title and abstract were independently screened by two reviewers to determine eligibility. Inclusion was only publications focused on CBCT imaging of bony injuries. We included studies describing patient cohorts (prospective or retrospective), although secondary reviews and meta-analyses were excluded to ensure the dataset was unique. Non-MSK CBCT applications such as dental, breast, head and neck, anthropology, or paleontology were discounted. Studies utilizing cadavers and phantoms are essential in medical imaging research; however, they were excluded from this review as their properties vary [23]. In addition, studies reporting on other imaging modalities or the CBCT technology applications in fluoroscopy, bone densitometry, or image-guided radiation therapy (IGRT) were also excluded. Conflicts at screening or full-text review were resolved through consensus [24]. Full-text review was then undertaken in Covidence, continuing the dual reviewer method.

### Data extraction

A custom-made data extraction tool was created in Covidence to collate relevant data. All features relevant to the scoping review aims were extracted independently by two reviewers. When necessary, the extraction process included arbitration by a third reviewer for consistency in consensus. Once complete, the data extraction tool was exported to Microsoft Excel for analysis. Quality assessment is not a required feature of scoping reviews and was not completed for the included articles, partly due to the diverse range of study designs.

## Results

**Fig. 1:**
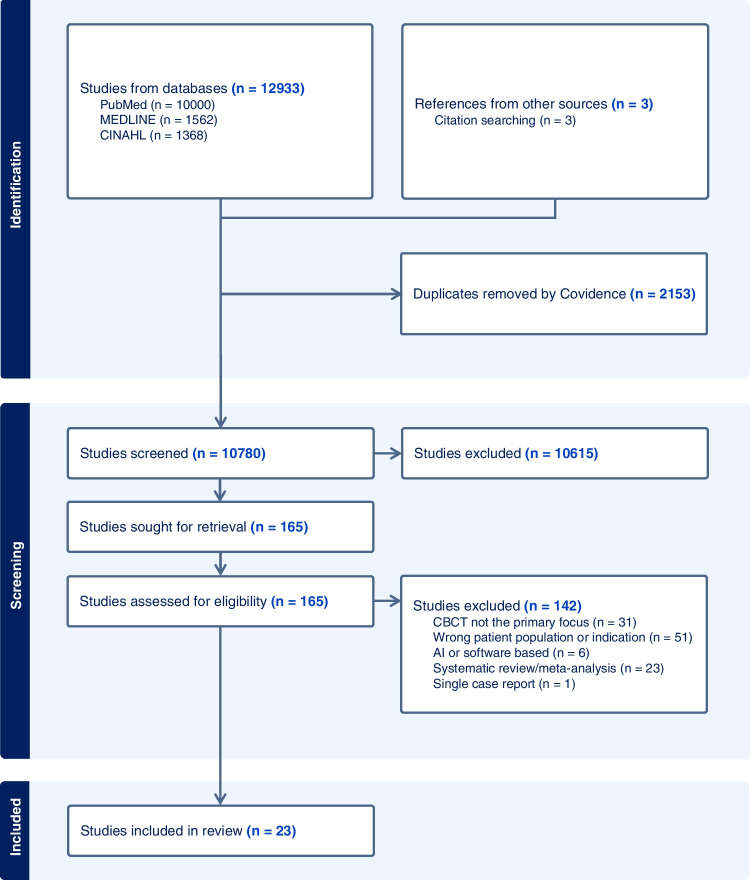
PRISMA flow chart detailing the identification, screening, and review process

The initial database search yielded 12,933 articles, of which 2153 were automatically identified as duplicates by Covidence and removed (Fig. 1). A total of 10,780 articles entered the title and abstract screening process, with 10,615 articles excluded at this stage. Exclusions at the full-text stage were primarily because CBCT was not the primary focus, or they were the wrong population type or indication. The remaining 23 articles were included in the review.

All 23 articles were single-center evaluations with 22 different lead authors from 18 centers across 11 countries (Table 1). European studies dominated the sample, although there was no correlation between the country of origin (based on lead author affiliation) and the CBCT system used. The PlanMed Verity scanner was used in half of the studies [5, 6, 25–34], with others utilizing the Carestream Onsight 3D [14, 35–37], Siemens Multitom Rax [38–40], Newtom 5G [41–43], and QR-DVT 9000 [44]. All studies included at least one radiologist author, with collaborating authors from a range of different imaging and clinical disciplines; however, only three of the articles were published in non-imaging–focused journals [30, 33, 37].

**Table 1:** Summary of the included studies (*n* = 23)

Study	Author specialism (s)	Lead author country	CBCT system	Comparison modality	Sample	study Design	Anatomy	outcomes
Allen et al. [25]	Radiology; research	UK	PlanMed Verity	US	100	Prospective observational study	Ankle	CBCT superior to radiographs for fracture detection
Borel et al. [26]	Radiology; orthopedics	France	PlanMed verity	MRI; radiographs	49	Prospective cohort study	Wrist	CBCT superior to radiographs for fracture detection
Bry et al. [35]	Radiology; medical physics	Finland	Carestream OnSight 3D	Radiographs	16	Prospective cohort study	wrist	CBCT superior to radiographs for fracture detection
Colville et al. [36]	Radiology; radiography	UK	Carestream OnSight 3D	Radiographs	101	Retrospective cohort study	wrist	CBCT superior to radiographs for fracture detection
DeSmet et al. [41]	Radiology	Belgium	Newtom 5G	Radiographs	216	Prospective cohort study	Ankle; elbow; foot; hand; knee; wrist	CBCT superior to radiographs for fracture detection
Dubreuil et al. [42]	Radiology; research; ENT	France	Newtom 5G	MSCT	36	Prospective cohort study	Ankle; foot; wrist	CBCT provides additional diagnostic information to MSCT at a lower dose
Edlund et al. [27]	Radiology	Sweden	PlanMed Verity	MRI; radiographs	95	Prospective cohort study	Wrist	CBCT superior to radiographs; however, MRI found additional injuries
Faccioli et al. [44]	Radiology	Italy	Newtom QR-DVT 9000	MSCT	57	Prospective cohort study	Hand	CBCT is effective for preoperative assessment
Falkowski et al. [38]	Radiology	Switzerland	Siemens Multitom Rax	MSCT	46	Prospective cohort study	Ankle; arm; foot; hand; leg; wrist	CBCT is an effective fracture diagnosis tool
Gibney et al. [28]	Radiology; orthopedics; medicine	Ireland	PlanMed Verity	MSCT; MRI; radiographs	166	Prospective cohort study	Wrist	CBCT is an effective imaging tool
Gibney et al. [29]	Radiology; orthopedics; ED	Ireland	PlanMed Verity	Radiographs	117	Prospective cohort study	Wrist	CBCT superior to radiographs for fracture detection
Grunz et al. [39]	Radiology; plastic surgery	Germany	Siemens Multitom Rax	Radiographs	92	Retrospective cohort study	Ankle; foot; hand; wrist	CBCT superior to radiographs for fracture detection
Huang et al. [6]	Radiology	USA	PlanMed Verity	MSCT; radiographs	50	Prospective cohort study	Ankle; elbow; foot; hand; knee; wrist	CBCT provided additional diagnostic information to MSCT at a lower dose
Jacques et al. [37]	Radiology; orthopedics	France	Carestream OnSight 3D	MSCT; radiographs	2390	Retrospective cohort	Ankle; elbow; foot; hand; knee; wrist	CBCT reduced overall dose and accelerated turnover
Krayem et al. [30]	Radiology; orthopedics; bioengineering	Sweden	PlanMed Verity	Radiographs	1058	Retrospective cohort	Wrist	CBCT superior to radiographs for fracture detection
Kunz et al. [40]	Radiology; plastic surgery	Germany	Siemens Multitom Rax	Radiographs	23	Retrospective cohort study	Elbow	CBCT is an effective fracture diagnosis tool
Lang et al. [31]	Radiology	Germany	PlanMed Verity	MSCT	68	Retrospective cohort study	Wrist	CBCT image quality is inferior to MSCT
Murphy et al. [32]	Radiography; medical physics	Ireland	PlanMed Verity	Ultra-low–dose CT	66	Prospective cohort study	Ankle; foot; hand; wrist	Standard dose CBCT is superior to ultra-low–dose CT for fracture diagnosis
Neubauer et al. [33]	Radiology; orthopedic surgery; plastic surgery	Germany	PlanMed Verity	Radiographs	102	Retrospective cohort study	Wrist	CBCT superior to radiographs for fracture detection
Ricci et al. 2019 [43]	Radiology; orthopedics	Italy	Newtom 5G	Radiographs	198	Retrospective cohort study	Ankle; elbow; foot; knee; wrist	CBCT superior to radiographs for fracture detection
Snaith et al. [14]	Radiography; radiology; ED	UK	Carestream OnSight 3D	No comparator	68	Prospective cohort study	Wrist	CBCT superior to radiographs for fracture detection
Suojärvi et al. [34]	Radiology; orthopedics	Finland	PlanMed verity	Radiographs	37	Prospective cohort study	Wrist	CBCT superior to radiographs for fracture detection
Tschauner et al. [5]	Radiology, pediatric surgery	Austria	PlanMed verity	MSCT	59	Prospective cohort study	Ankle; elbow; foot; hand; knee; wrist	CBCT had lower radiation dose with better image quality

*ED* emergency department, *CBCT* cone beam computed tomography, *MSCT* multislice computed tomography, *MRI* magnetic resonance imaging, *US* ultrasound

One study was considered observational, as it compared CBCT with ultrasound in the context of both occult fracture and ligament injury diagnosis [25], whereas 14 included the CBCT scan as an additional intervention within a prospective cohort study. Sample sizes varied from 1 to 2390, with most including both male and female patients with no restriction on age, although one focused solely on pediatric imaging [5].

The majority of the articles included wrist imaging, with 50% of articles solely investigating radiocarpal trauma. Although some studies included the lower limb, no axial skeletal imaging was found in this review.

Diagnostic accuracy was an overarching concept within the identified articles and was described in 22 out of the 23 studies. Multiple modality comparisons were utilised in six studies, with three studies including MRI [14, 26–28]. Radiographs were the initial imaging modality in 17 studies, confirming that in many centers, this remains the first-line diagnostic tool for bony injuries. Comparing CBCT with radiographs was the most common approach, with 15 studies performing this comparison. Five studies used MSCT as the preliminary imaging modality [5, 31, 38, 42, 44], with a single center using CBCT as the first-line test [32]. Most studies compared CBCT with standardized MSCT protocols, although one explored the option to use an ultralow dose MSCT protocol as a comparator to CBCT [32]. In addition, radiation dose differences between ionising radiation imaging modalities were explored in five articles within the sample [6, 37, 38, 40, 41], and a further five studies investigated image quality between modalities [5, 6, 26, 31, 38].

## Discussion

In this scoping review, only 23 articles were identified regarding the utilization of CBCT within the context of MSK trauma in the human population. This highlights the lack of research evidence for the utilization of this technology in practice, likely because CBCT is still viewed as a novel imaging modality and access to this technology is more limited. Positively, the majority of studies investigating CBCT within trauma pathways did so through prospective cohort studies, involving the recruitment of eligible patients.

### Scanner technology

The limited field of view and anatomical coverage associated with CBCT technology likely have influenced the focus to single-joint injuries rather than larger volumes. A range of scanner manufacturers were included in the research, although the gantry-free CBCT technology, for instance, the Siemens Multitom Rax, was least evident. When utilizing this scanner model, Kunz et al. [40] concluded that the system was reliable and effective for the detection and exclusion of fractures, including the articular involvement and multi-fragmentary pattern determination. This is further supported by Grunz et al. [39], who concluded that this type of scanner was successful for detecting small bone fractures, articular involvement, and joint trauma. Due to the small number of studies using this type of CBCT system, the range of evidence may not be extensive enough to draw valid conclusions.

In contrast, the PlanMed Verity was the most commonly included system, with just over half of the published studies using this model. It is not evident why this scanner is used more frequently; however, both Gibney et al. [28] and Edlund et al. [27] confirmed that the images they evaluated showed a high sensitivity for fracture detection, but did not suggest this was a result of the model.

The use of weight-bearing imaging is significant in the development of research evidence regarding CBCT; however, no weight-bearing studies were included in this review. This may be that weight-bearing imaging may not be seen as wholly relevant within acute trauma imaging, due to limited patient ability and discomfort. That said, weight-bearing imaging may be advantageous when investigating soft tissue pathologies.

### CBCT applications

CBCT was been found to be effective in the assessment of hand and wrist injuries. Gibney et al. [29] found that 50% of patients with a negative initial radiographic examination had an acute fracture identified on CBCT images. Further, Colville et al. [36] concluded that CBCT has a higher sensitivity for the detection of radiocarpal injuries when compared with radiography. The value of CBCT within radiography-occult fracture diagnosis was a recurring theme with many of the articles describing CBCT as a reliable diagnostic tool for injuries not evident on the initial imaging investigation. For example, Snaith et al. [14] found that CBCT imaging confirmed radiographic occult fractures and importantly identified additional fractures not previously suspected. To further evidence this, Borel et al. [26] determined that CBCT is a reliable diagnostic tool for the investigation of radiographically occult fractures of the scaphoid as well as occult cortical fractures of the entire wrist anatomy. Colville et al.’s [36] findings reinforce the opportunity to detect radiographically occult fractures, with CBCT use resulting in the diagnosis of 26 additional fractures within their cohort. This range of evidence promotes the idea that CBCT imaging would be a successful follow-up imaging method for those who have had negative initial radiographs, but where pain and discomfort persist. Suojärvi et al. [34] suggested that CBCT could be regarded as a low-dose preoperative assessment imaging technique, especially in the wrist, where it was confirmed to have a high level of agreement for measures of intra-articular fractures. In addition, CBCT has been suggested to be advantageous for preoperative assessment in finger fractures, with Faccioli et al. [44] concluding that CBCT had a 100% sensitivity and specificity for evaluation of phalangeal articular surface involvement.

Evidence of the utilization of CBCT for lower extremity bony trauma is limited. Allan et al. [25] suggested that CBCT could be an acceptable replacement for radiographs in the examination of acute ankle injuries. Ricci et al. [43] also concluded that CBCT could be an important diagnostic instrument for foot fractures, particularly complex midfoot injuries. However, further evidence regarding lower extremity CBCT imaging is necessary to gain a comprehensive understanding of the opportunities afforded by this modality. In addition, no research surrounding other anatomical areas, such as the shoulder or hip, was identified within this review.

### Comparison across imaging modalities

Many of the selected studies compared CBCT with radiographs, and multiple articles concluded that CBCT is superior to radiography in fracture detection. For instance, Huang et al. [6] found that the improved image quality led to better visualization of fractures, concurring with Krayem et al. [30] who found twice as many scaphoid fractures using CBCT compared with radiographs. That said, one study suggested that CBCT image quality was inferior to MSCT [44]. Jacques et al. [37] suggested that the implementation of CBCT enabled higher patient throughput, implying that utilization can increase efficiency. In addition, they stated that CBCT would decrease patient radiation dose, aligning with Huang et al.’s [6] findings, that it provided more diagnostic information than MSCT at a lower dose.

A number of authors identified limitations regarding the use of CBCT. Lang et al. [31] noted that motion artefacts were more frequently seen in CBCT than MSCT, and the overall differences in image quality favored MSCT, in particular regarding soft tissue depiction. Similarly, Facciolli et al. [44] stated that CBCT was less accurate when compared with MSCT in depicting small bone fragments, but it can still be considered a reliable imaging tool. The limitations identified are not discussed in any detail and require further investigation across a larger scale of research.

### Implications for practice

Regarding clinical practice, the value and potential of CBCT as a diagnostic tool within extremity trauma pathways is clear. However, future considerations must include the identification of staff training requirements and determining safe staffing numbers, as there are evident deficiencies within the current literature. MSCT is well established within major trauma and complex case identification, whereas CBCT is frequently only utilized for extremity imaging. Therefore, there are likely to be fewer intensive training requirements, but additional research to develop a staff training strategy and competencies could aid future integration into clinical practice.

Regarding the economic value and cost-effectiveness of CBCT imaging, there is limited evidence surrounding the actual running costs. Borel et al. [26] suggested that along with the clinical advantages of CBCT, there is also a relatively small outlay in the purchase of the equipment compared with MSCT or MRI. Throughout most of the selected articles, no economic analysis was performed; therefore, knowledge regarding this concept is insufficient. As the only study identified outside this review, economic modelling has shown that CBCT can be cost-saving in comparison with MSCT, in this case in the management of finger fractures [45]. As CBCT provides a reliable, precise diagnosis, additional diagnostic tests are often not required, and early diagnosis can lead to reduced hospital visits [14]. The results may lead to an overall cost saving.

In order to consider the implementation of CBCT in practice, it is appropriate to understand the potential for wider applications outside of the remit of this scoping review. Beyond the initial investigation of bony trauma, CBCT has a role in fracture healing assessment but also in investigation of bone lesions, infection, malalignment, and degenerative disease [12, 14]. In particular, the opportunities associated with the weight-bearing capabilities of some of the scanners afford the unique opportunity to assess functional alignment of the lower limb. This has been shown to be useful in foot and ankle pathology [46], with a recent economic assessment suggesting that the technology could save time and costs [47]. The evidence for weight-bearing imaging of the knee is growing both in terms of joint assessment [46, 48] and in arthrography, both in the knee [14, 49] and elsewhere [50–52]. Additionally, the development of large bore CBCT scanners is now enabling investigation of the spine and hip in addition to the extremities [53].

### Areas for further research

Overall, there is more research needed to evidence the potential of CBCT to improve MSK trauma pathways. The reviewed literature suggests that CBCT is effective for the evaluation of wrist trauma; however, there is a deficit of published research across other anatomical areas. Further, research regarding inherent CBCT artefacts, including motion and from metalwork, requires greater understanding.

A number of limitations must be considered within this review. Only studies published in English were included, and only the lead author was reported in the context of geography. Varied interventions, populations, and outcomes were present across the sample, and the differences in study design limit the ability to directly compare outcomes; therefore, no meta-analysis was possible given the differences in data. Only including human studies may mean that potentially valuable cadaver and phantom studies were excluded. Finally, only bony injuries were investigated within this scoping review, and no attempt was made to explore soft tissue or ligamentous injuries. In addition, only malalignment where it relates to bony injury rather than functional abnormalities has been considered. It is acknowledged that soft tissue injuries are relevant in trauma assessment, and therefore, further assessment of this topic is required.

In conclusion, this review sought to map the existing literature surrounding CBCT for MSK trauma. The published literature regarding the use of CBCT in MSK imaging demonstrates the growing number of opportunities in this area. Numerous cohort studies support the use of the technology for high-resolution 3D imaging of the extremities, particularly in fracture detection and characterization when compared with radiographs; however, there is less evidence of CBCT’s role in lower limb trauma, alongside an absence of economic and resource considerations. Although there is variability across systems and imaging protocols, the value of CBCT within the diagnosis of occult fractures and preoperative assessment has been illustrated. Awareness of the advantages and limitations of CBCT when compared with other modalities is urged, although likely to evolve. Furthermore, evaluation of the impact of CBCT on clinical pathways and patient management and their long-term outcomes requires further investigation.

## Data Availability

Not applicable.
